# Significance of occult neoplastic cells on tumor metastasis: a case report of gastric cancer

**DOI:** 10.1186/1746-1596-5-14

**Published:** 2010-02-24

**Authors:** Shinkichi Sato, Masaya Mukai

**Affiliations:** 1 Department of Pathology, Tokai University Oiso Hospital, 21-1 Gakyou, Oiso, Kanagawa 259-0198, Japan; 2 Department of Surgery, Tokai University Hachioji Hospital, 1838 Ishikawa-cho, Hachioji, Tokyo 192-0032, Japan

## Abstract

**Background:**

Occult neoplastic cells (ONCs) are the tumor cells floating in the lymph node sinuses, distant from the primary tumor, and supposed to be one of most reliable marker of prognosis.

**Methods:**

We report here the case of a 52-year-old woman with a gastric cancer associated by numerous ONCs.

**Results:**

Postoperative examination of the stomach disclosed an advanced, poorly differentiated adenocarcinoma with frequent lymph node metastases. In addition to ONCs and occasional micrometastases, focal aggregates of ONCs, one of the possible intermediate lesions between the ONCs and the usual metastases, are also observed.

**Conclusions:**

In the present case, at least some of ONCs seem to form the microaggregates of tumor cells in lymph nodes, anchor in the sinuses, and grow up to the large tumorous lesion. Even if most of the ONCs were trapped and disappeared under the influence of tumor immunity, the detection of ONCs could be one of the reliable clues to estimate the prognosis.

## Background

Level of malignancy in cancer is defined by the speed of proliferation and the ability of invasion/recurrence/metastasis, and at least partially depends on the histological type of primary tumor. Postoperative pathological examination of primary tumor discloses the histological type, degree of invasion, lymphatic and venous involvement and the lymph node metastasis, and these factors are considered to contribute the possibility of postoperative recurrence/metastasis. Accurate prospect of the prognosis could be the strong support for choosing postoperative therapy.

It is thought that fatal recurrence/metastasis of the cancer cases occurs in the liver or lungs after surgical resection when free tumor cells or cell clusters circulate through the body during the perioperative period and escape the host immune system, survive and proliferate in these organs [1,2]. The detection of these free tumor cells or cell clusters is considered to be the useful marker for the evaluation of the prognosis.

Occult neoplastic cells (ONCs) are the tumor cells freely floating in lymph node sinuses distant from the primary tumor and the close relationship to the recurrence/metastasis of various malignancies has been suggested [1-5]. Microaggregates of tumor cells have also been considered as one of the most possible cause of the metastasis and are easily detected by routine Hematoxylin & Eosin (H&E) stain, though ONCs are hardly detected. Immunohistochemical technique using the epithelial marker makes it easy to detect solitary, floating tumor cells in the lymph node sinuses [1-5].

We report here a case of gastric cancer with many ONCs in dissected lymph nodes as well as microaggregates, the possible intermediate lesions to the metastasis, by immunohistochemical study.

## Case report

A 52 year-old woman, a housewife, was admitted to Tokai University Oiso Hospital because of abdominal discomfort and back pain. She had no past history of malignancy. The family history was non-contributory. Gastric endoscopy showed an irregularly outlined, centrally ulcerated mass at the lesser curvature of mid body to antrum of the stomach. Biopsy specimens taken from the tumor indicated poorly differentiated adenocarcinoma mainly composed of signet ring cell carcinoma. One month later, total gastrectomy, regional lymph node dissection and following chemotherapy by 5FU were performed. Post-operative evaluation was T3N2M0. Two years after surgery, abdominal mass involving the umbilicus and transverse colon became evident. Biopsy specimen taken from the mass revealed the metastatic foci of adenocarcinoma. Ascites also contained adenocarcinoma cells. She was expired because of systemic metastases two years and eight months after the surgery.

For light microscopy, the specimen was fixed in 10% buffered formalin, and 4 mm-thick tissue slices were embedded in paraffin. Paraffin sections were stained with HE.

Immunohistochemical detection of cytokeratin in harvested LN was performed by the indirect immunoperoxidase method using a monoclonal anti-cytokeratin antibody (AE1/AE3; Fuji Chemical Industries, Ltd., Japan) [6,7].

## Pathological Findings

**Figure 1:**
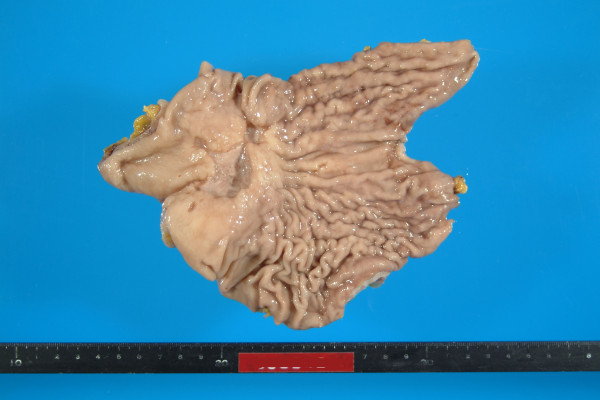
**Stomach with the ulcerative tumor**.

**Figure 2:**
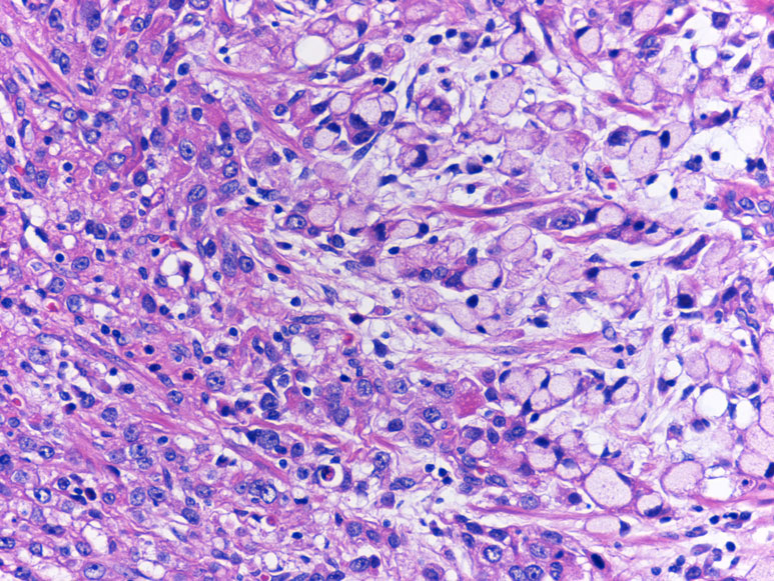
**Poorly differentiated adenocarcinoma with signet ring type tumor cells (H&E)**.

On gross pathology the resected specimen contained a centrally ulcerated mass (40 × 50 × 20 mm) at the lesser curvature to anterior wall of the stomach (Fig. 1). Microscopically the tumor was composed mainly of signet ring type tumor cells in the mucosa and of poorly differentiated adenocarcinoma cells with moderate to severe cytological atypia in the submucosa to the serosa (Fig. 2), arranged in trabecular or abortive tubular structures with stromal fibrosis. Dissected lymph nodes contained metastatic deposits of adenocarcinoma in 6 out of 22 nodes.

**Figure 3:**
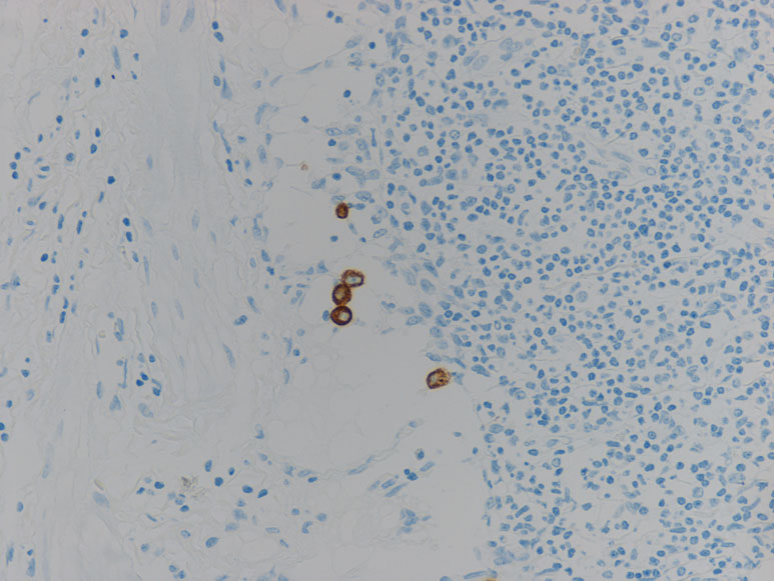
**Floating tumor cells in lymphatic sinus (Cytokeratin AE1/AE3)**.

**Figure 4:**
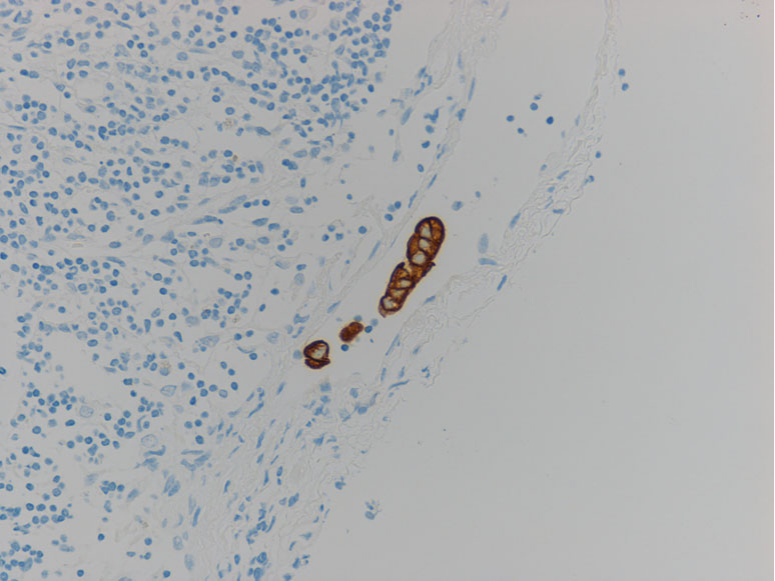
**Floating tumor cell islands in lymphatic sinus (Cytokeratin AE1/AE3)**.

**Figure 5:**
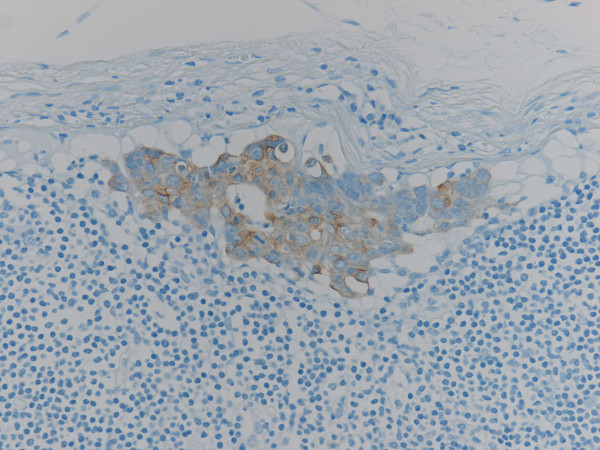
**Micrometastasis anchoring in the lymphatic sinus (Cytokeratin AE1/AE3)**.

**Figure 6:**
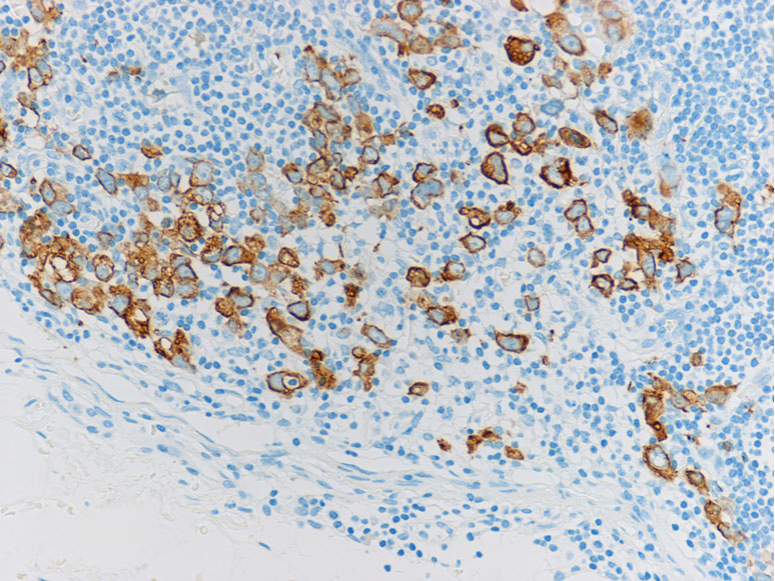
**Floating tumor cells aggregated in the lymph node (Cytokeratin AE1/AE3)**.

Immunohistochemical study for cytokeratin (AE1/AE3) revealed diffuse and strongly positive reaction in the cytoplasm of normal epithelial and carcinoma cells in the primary lesion of the stomach, and also in the metastatic lesions in the lymph nodes and abdominal mass. In addition to the metastatic deposits of adenocarcinoma easily identified by HE stain in lymph nodes, tiny aggregates of tumor cells and single atypical tumor cells floating in the sinuses, that were negative for metastasis by routine H&E staining, are also identified by immunohistochemical detection (Fig. 3). There were a few patterns of tumor cells in the lymph node sinuses: i) single tumor cell; ii) a tiny aggregate of several tumor cells (Fig. 4); iii) the larger aggregate of tumor cells anchored in the sinuses, called "micrometastasis"(MM) (Fig. 5); iv) group of tiny tumor cell aggregates embedded in the sinus wall and lymphocytes (Fig. 6).

## Discussion

The close relationship between ONCs and the recurrence/metastasis of various malignancies has been suggested [1-5]. In this case, many ONCs and micrometastases are identified in the lymph nodes at the operation and the recurrence and distant metastasis of tumor became evident two years later. Although no direct demonstration could be made, the possible role of ONCs are suggested in this case too.

In cancer cases, some viable tumor cells are considered to invade into the lymphatics or veins from the primary tumor and to disseminate throughout the body [4], though most of these tumor cells are disposed under the host immune system [8,9]. Remaining tumor cells pass through the defense system and make the metastatic foci in distant organs. It has been reported that cancer cells have a high affinity for sites of vascular endothelial damage, and tumor cells that lodge in a particular organ gradually infiltrates into the parenchyma due to the action of fibrinolysis/coagulation factors and/or vascular endothelial growth factor (VEGF)[10,11]. Most likely cause of recurrence/metastasis after curative surgery is the proliferation of residual cancer cells anywhere in the body of the host. Many studies have shown a close relationship between the ONCs floating in the lymph node sinuses and the recurrence/metastasis of various malignancies, such as cancer of the breast, lung, esophagus, stomach, and large bowel [1-5]. It is unclear whether the ONCs could be a marker of the tumor cell metastasizing ability or the ONCs would gather to be a clot, anchor and form micrometastasis (MM) to lead the usual metastatic lesion. In the present case, ONCs are focally observed in groups and these features are considered to be one of the process to make a MM. Although the relationship between ONCs and MM is unclear, morphometric measurement of MIB-1 index disclosed almost similar levels, 20% to 40%, in the primary tumor, MM and ONCs, suggestive of similar proliferating ability. On the other hand, in terms of the vulnerability, the groups of tumor cells, especially closely adhered tumor cells, MM, are considered to be stronger than a single tumor cell against the nutritional damage or various kind of attacks, for example anti-cancer drug or tumor immunity. Non-adhered ONCs in groups, focally observed in this case, are also suggestive of one of the processes to be a MM.

Role of MM in the lymph nodes or bone marrow has been reported in breast cancer [12-16], though less clear in colorectal cancer and no consensus in other malignancies such as cancer of the lung, esophagus and stomach [2-4]. MM should be considered to be macroscopically invisible and microscopically detectable lesions by routine H&E stain. Complete resection of MM by lymph node dissection has been reported to relate the good prognosis without recurrence, while residual MM may lead to the recurrence or metastasis [1,8,9].

ONCs are circulating cancer cells and can be identified by immunohistochemical technique after being trapped in lymph nodes surrounding the primary tumor. These cells are considered to be associated with occult systemic metastasis

## Conclusions

In the present case, at least some of ONCs seem to form the microaggregates of tumor cells in lymph nodes, anchor in the sinuses, and grow up to the large tumorous lesion. Even if most of the ONCs were trapped and disappeared under the influence of tumor immunity, the detection of ONCs could be one of the reliable clues to estimate the prognosis.

## Consent

Written informed consent was obtained from the patient for publication of this case report and accompanying images. A copy of the written consent is available for review by the Editor-in-Chief of this journal.

## Competing interests

The authors declare that they have no competing interests.

## Authors' contributions

SS drafted the manuscript and described the pathology component and took photographs, participated in writing the discussion, coordinated and edited the manuscript. MM edited the clinical part of the manuscript. MM provided the clinical data and edited the clinical case presentation. SS reviewed the entire manuscript, participated in writing the discussion and the pathology component and edited the manuscript. All authors read and approved the final manuscript.
